# Comparison of stool versus rectal swab samples and storage conditions on bacterial community profiles

**DOI:** 10.1186/s12866-017-0983-9

**Published:** 2017-03-31

**Authors:** Christine M. Bassis, Nicholas M. Moore, Karen Lolans, Anna M. Seekatz, Robert A. Weinstein, Vincent B. Young, Mary K. Hayden, for the CDC Prevention Epicenters Program

**Affiliations:** 1 Department of Internal Medicine, Division of Infectious Diseases, University of Michigan, Ann Arbor, MI 48109 USA; 2 Department of Medical Laboratory Science, Rush University Medical Center, Chicago, IL 60612 USA; 3 Department of Medicine (Infectious Diseases), Rush University Medical Center, Chicago, IL 60612 USA

**Keywords:** 16S rRNA gene sequences, Microbiota, Gastrointestinal tract, Stool, Rectal swab, Microbial community, Gut microbiota

## Abstract

**Background:**

Sample collection for gut microbiota analysis from in-patients can be challenging. Collection method and storage conditions are potential sources of variability. In this study, we compared the bacterial microbiota from stool stored under different conditions, as well as stool and swab samples, to assess differences due to sample storage conditions and collection method.

**Methods:**

Using bacterial 16S rRNA gene sequence analysis, we compared the microbiota profiles of stool samples stored and collected under various conditions. Stool samples (2 liquid, 1 formed) from three different patients at two hospitals were each evaluated under the following conditions: immediately frozen at -80°C, stored at 4°C for 12-48 hours before freezing at -80°C and stored at -20°C with 1-2 thaw cycles before storage at -80°C. Additionally, 8 stool and 30 rectal swab samples were collected from 8 in-patients at one hospital. Microbiota differences were assessed using the Yue and Clayton dissimilarity index (θ_YC_ distance) and analysis of molecular variance (AMOVA).

**Results:**

Regardless of the storage conditions, the bacterial communities of aliquots from the same stool samples were very similar based on θ_YC_ distances (median intra-sample θ_YC_ distance: 0.035, IQR: 0.015-0.061) compared to aliquots from different stool samples (median inter-sample θ_YC_ distance: 0.93, IQR: 0.85-0.97) (Wilcoxon test *p*-value: <0.0001). For the stool and rectal swab comparison, samples from different patients, regardless of sample collection method, were significantly different (AMOVA *p*-values: <0.001-0.029) compared to no significant difference between all stool and swab samples (AMOVA *p*-value: 0.976). The θ_YC_ dissimilarity index between swab and stool samples was significantly lower within individuals (median 0.17, IQR: 0.10-0.27) than between individuals (median 0.93, IQR: 0.85-0.97) (Wilcoxon test *p*-value: <0.0001), indicating minimal differences between stool and swab samples collected from the same individual over the sampling period.

**Conclusion:**

For gastrointestinal microbiota studies based on bacterial 16S rRNA gene sequence analysis, interim stool sample storage at 4 °C or -20 °C, rather than immediate storage at -80 °C, does not significantly alter results. Additionally, stool and rectal swab microbiotas from the same subject were highly similar, indicating that these sampling methods could be used interchangeably to assess the community structure of the distal GI tract.

## Background

The diverse communities of microorganisms that compose the human gut microbiota play key roles in health and disease. Advances in sequencing technology have facilitated the wide use of bacterial 16S rRNA-encoding gene sequence analysis for the identification of bacterial lineages as well as their relative abundances in microbial communities. Alterations in the gut microbiota are associated with numerous diseases including cardiovascular disease, inflammatory bowel diseases (IBD) and colorectal cancer as well as increased susceptibility to infections [1–5]. Carriage of multidrug-resistant opportunistic enteric pathogens, such as vancomycin-resistant enterococcus (VRE) and extended spectrum beta-lactamase (ESBL) producing Enterobacteriaceae, has also been associated with changes in intestinal bacterial communities among hospital patients [6]. This observation has resulted in an interest in understanding the gastrointestinal microbiota features that may predispose patients to colonization with multidrug-resistant organisms (MDROs), which could lead to the development of interventions to prevent MDRO colonization and subsequent infection.

Much of the work in gastrointestinal microbiota analyses from human subjects has been done using stool samples (e.g. [7]). In hospitals, patient-level factors, such as fecal incontinence, and facility-level factors, such as heavy nursing workloads, can make collection of freshly passed stool challenging or impractical. In contrast, collection of rectal swab samples for surveillance cultures among hospitalized patients to determine colonization with MDROs is a routine infection control practice [8–12].

Fecal samples for routine culture are often preserved using chemicals, refrigeration, or freezing depending on the testing that will occur on the specimen. In microbiota analyses, it is important to utilize a procedure that will minimize DNA alteration in samples prior to the analysis. If DNA extraction is not done immediately after collection, the gold standard is to store specimens at -80 °C. In some clinical settings, however, there may not be immediate access to an ultralow-temperature freezer, and samples may need to be transported at higher temperatures before reaching the lab.

In this study, we compared the bacterial profiles of rectal swab samples to stool samples collected from patients at one hospital. We also assessed the effects of common storage conditions on the composition of the fecal microbiota. The objective of our study was to determine the effects of storage and sampling method on the gastrointestinal microbiota.

## Results

### Evaluation of stool storage conditions

We first compared the effects of different storage conditions on the microbial composition of stool samples (collected into a sterile container without the use of a preservative) from three patients. Sample A was a diarrheal stool from a patient who had been in Hospital A for 11 days. The patient was currently receiving enteral nutrition via a gastrointestinal tube and had received intravenous colistin, daptomycin, and vancomycin. Sample B was a clear, watery stool from a patient who had been hospitalized for seven days at Hospital B. The patient had received 7 doses of oral levofloxacin and 1 dose of intravenous vancomycin. Sample C was a formed stool from an outpatient at Hospital B who had completed a two-week course of oral clarithromycin and metronidazole approximately 2 months prior to sample collection.

Each sample was split into 15 aliquots and tested in triplicate under the 5 storage conditions described in Table 1. For stool samples, dilution of DNA often improves PCR amplification. So, in addition to comparing storage conditions, we also tested the effect of dilution on community analysis by comparing DNA from the same samples that was undiluted and diluted 1:10 for the PCR. We then used 16S rRNA gene sequence analysis to assess the microbial community profile of each sample condition. Aliquots that did not amplify or were poorly sequenced (<1000 sequences per sample) were not included in the analysis. After sequence processing we obtained 1,882,278 sequences from the V4 region of the 16S rRNA gene from 74 sample aliquots with an average of 25,436 ± 10,999 (SD) sequences per sample.

**Table 1:** Stool temperature storage conditions

Storage Condition Number (SC#)	Temperature Conditions
1	Immediately frozen -80 °C
2	Held overnight at 4 °C, then frozen -80 °C
3	48 h at 4 °C, then frozen -80 °C
4	Immediately frozen -20 °C for 24 h, 1 thaw cycle, frozen at -80 °C
5	Immediately frozen -20 °C for 24 h, 1^st^ thaw, frozen -20 °C, 2^nd^ thaw, frozen at -80 °C

**Fig. 1:**
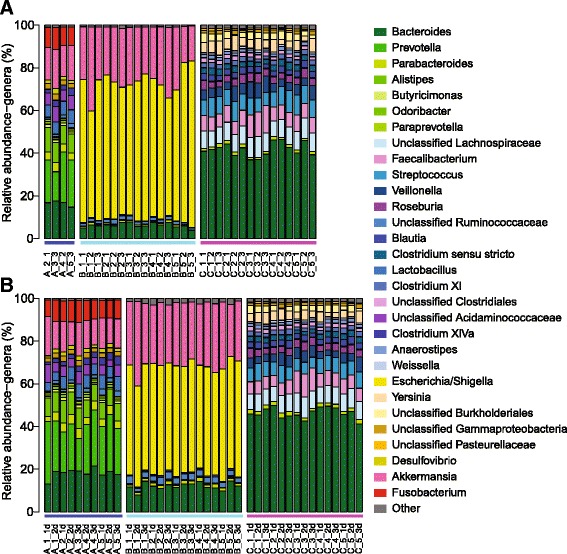
Bacterial community composition of stool samples subjected to various temperature storage conditions. The relative abundances of 16S rRNA gene sequences (V4 region), classified to the genus level when possible, are shown. Labels indicate sample, storage condition (SC) as described in Table 1 and aliquot in the following format: sample_SC#_aliquot#. For example, A_2_1 is from sample A, storage condition number 2, aliquot number 1. The colors of the horizontal bars above labels correspond to sample color-coding in Fig. 2a. **a** Undiluted DNA for PCR. **b** DNA diluted 1:10 for PCR, indicated by d after aliquot number

**Fig. 2:**
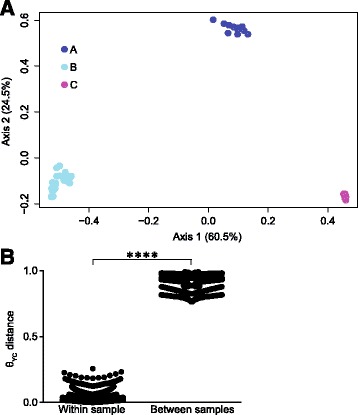
θ_YC_ distances between bacterial communities of stool sample aliquots subjected to various temperature storage conditions from 3 patients. **a** Principal coordinates analysis (PCoA) of θ_YC_ distances between bacterial communities of stool sample aliquots. The aliquots of each sample were represented by a different color which corresponds to the colors of the horizontal bars above labels in Fig. 1. **b** The θ_YC_ distances between aliquots was significantly lower within samples (median: 0.035, IQR: 0.015-0.061) than between samples (median: 0.93, IQR: 0.85-0.97) (Wilcoxon test *p*-value: <0.0001)

The bacterial community composition was not strongly affected by storage condition or DNA dilution (Fig. 1). However, the gut microbiota of each patient was significantly different from that of the other patients based on θ_YC_ distances (AMOVA p-value: <0.001 for all comparisons) (Fig. 2a). Although each patient displayed a distinct microbiota, community structure was markedly similar within each patient for all storage conditions and dilutions (Figs. 1 and 2). Regardless of storage condition or dilution, θ_YC_ distances between microbiota of aliquots from the same samples (median: 0.035, IQR: 0.015-0.061) were significantly lower than θ_YC_ distances between microbiota of aliquots from different samples (median: 0.93, IQR: 0.85-0.97) (Wilcoxon test p-value: <0.0001) (Fig. 2b). Additionally, overall community richness (the number of OTUs per sample) did not differ significantly between different storage conditions or dilutions within each patient (Kruskal-Wallis test) (OTUs per sample: A median: 89, IQR: 83-99.8; B median: 62.5, IQR: 58-70.8; C median: 112.5, IQR: 107-118.8). Our results indicate high similarity between the bacterial communities of aliquots from the same sample even when different storage conditions or dilutions were used.

### Rectal swab compared to stool specimens

To evaluate stool versus swab sample collection, we collected one stool sample and multiple rectal swabs during the next 24 to 27 h from 8 patients each: 6 women and 2 men, with a median age of 55 years (IQR: 47-58 years). To detect possible contamination, an unused swab and a reagents only/no sample control were processed through DNA isolation and PCR with the stool and swab samples. These control samples did not yield a PCR product that was visible on a gel (data not shown). After sequence processing we obtained 754,371 sequences from the V4 region of the 16S rRNA gene from 8 stool specimens and 30 swab samples with an average of 19,852 ± 8484 (SD) sequences per sample.

**Fig. 3:**
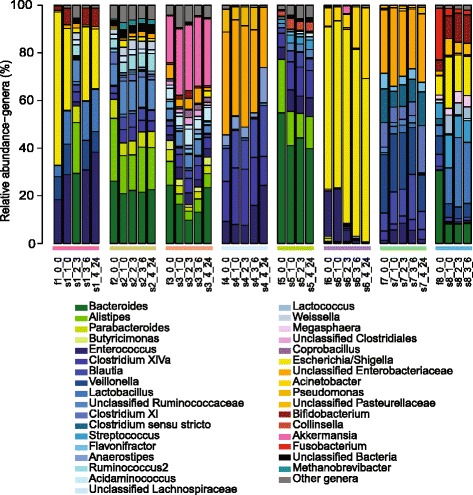
Bacterial community composition of stool and subsequent rectal swab samples. The relative abundances of sequences classified to the genus level when possible. Labels indicate sample type (f = stool, s = swab), subject number, sample number and approximate sampling time in hours relative to stool sample collection. For example, s1_4_24 indicates a swab sample from subject 1, sample number 4, collected at approximately 24 h after the stool sample. Please note that sample s1_2_3 is distinct from other subject 1 samples, likely due to contamination from a subject 2 sample. The colors of the horizontal bars above labels correspond to subject color-coding in Fig. 4a

**Fig. 4:**
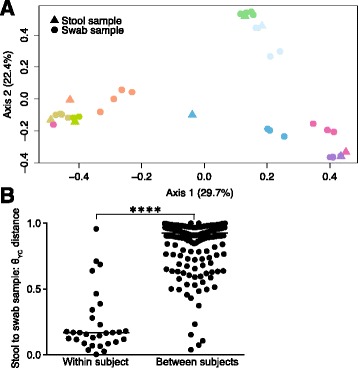
θ_YC_ distances between bacterial communities of stool and swab samples. **a** Principal coordinates analysis (PCoA) of θ_YC_ distances between bacterial communities of stool and swab samples. Samples are color-coded by subject. Shape indicates sample type: triangles = stool samples and circles = swab samples. **b** The θ_YC_ distances between swab and stool samples was significantly lower within subjects (median: 0.17, IQR: 0.10-0.27) than between subjects (median: 0.93, IQR: 0.85-0.97) (Wilcoxon test *p*-value: <0.0001)

Overall, the bacterial community structure was similar between the freshly passed stool and the rectal swabs collected at various time points within each patient (Figs. 3 and 4). PCoA of θ_YC_ distances indicated that the microbiota of stool samples and rectal swabs clustered by subject (Fig. 4a). In addition, regardless of sample collection method, there were significant differences between all subjects (AMOVA p-value: <0.001-0.029) compared to no overall difference between all stool and swab samples from within subjects (AMOVA p-value: 0.976). The θ_YC_ distance between swab and stool samples was significantly lower within subjects (median: 0.17, IQR: 0.10-0.27) compared to between subjects (median: 0.93, IQR: 0.85-0.97) (Wilcoxon test p-value <0.0001) (Fig. 4b). These results indicate that there were minimal differences present between stool and swab samples collected from the same subject over the sampling period up to 27 h after the baseline bowel movement.

Sample s1_2_3 from subject 1 was found to be an outlier, as the bacterial community profile does not correlate with other specimens from subject 1 (Figs. 3 and 4). We attempted to confirm the bacterial community composition of s1_2_3 from the second swab head of the dual swab sample. DNA isolation from the second swab head of s1_2_3 yielded no detectable DNA and PCR of the V4 region of the bacterial 16S rRNA gene did not yield a detectable product (data not shown), suggesting that contamination from another sample may explain these results.

## Discussion

Understanding links between the gastrointestinal microbiota and health in the clinical setting has the potential to improve patient care in the context of infection prevention and beyond. Optimizing study feasibility without altering results is critical for research on the gut microbiota in hospitalized patients. In this study we investigated the effects of sample storage conditions and collection methods on analysis of the gut microbiota.

Accurate analysis of the gastrointestinal microbiota based on bacterial 16S rRNA gene sequences did not require immediate storage of samples at -80 °C. Interim storage (24-48 h) of stool aliquots at temperatures likely to be available in a hospital (4 °C or -20 °C), even with 1 or 2 freeze/thaw cycles, didn’t significantly alter the microbiota. This agrees with a recent study showing minimal changes in the microbiota of stool samples stored without a preservative at -20 °C or 4 °C up to 8 weeks, although fungal growth is a likely complication with extended periods at 4 °C [13]. Storage at warmer temperatures have been shown to alter microbiota from stool [13, 14] and sputum [15].

The utility of rectal swab cultures for the surveillance of MDROs among hospitalized patients is widely recognized. Rectal swabs are relatively simple samples to collect, require no patient preparation, and can be transported easily from the bedside to the laboratory. In the hospital setting, which often includes medically complex patients and heavy nursing workloads, rectal swabs are more convenient to collect than stool samples. With healthier subjects, swabs can be self-collected with minimal instruction. In our study, we found bacterial communities in individual patients to be highly similar from stool and rectal swab samples. The overall composition of bacterial communities was comparable to a freshly passed stool specimen even in swabs collected up to 27-h after stool passage. A potential limitation of using swab samples compared to stool samples is that the amount of sample collected is smaller. In our study, successful bacterial community analysis was possible from most (29/30) of the rectal swab samples. One rectal swab sample yielded DNA levels too low for accurate bacterial community analysis, likely rendering that sample especially susceptible to contamination. Our findings suggest that rectal swabs are an acceptable and practical proxy for the collection of fecal specimens for stool microbiota analysis. Similarly, a previous study found that rectal swabs were a suitable alternative to stool for analyzing the intestinal microbiota using IS-pro, a method that differentiates bacteria based on internal transcribed spacer (ITS) length and phylum-specific fluorescent primers [16].

## Conclusions

Gastrointestinal microbiota studies based on bacterial 16S rRNA gene sequencing have options for interim sample storage conditions (4 °C or -20 °C vs. -80 °C) and sample collection methods (stool vs. rectal swab) that may increase sampling feasibility in the hospital setting without altering results.

## Methods

### Specimen selection and collection

To assess the effects of different storage conditions on bacterial community profiles, salvaged stool samples submitted to the clinical microbiology laboratory of a 108-bed long-term acute care hospital (hospital A) and a 720-bed tertiary, short-stay acute care hospital (hospital B) in Chicago, IL were tested. To compare rectal swabs with freshly passed stool, a convenience sample of subjects from hospital A (2 women, 6 men) was selected from those patients who were present in the facility on the day of sample collection. Stool that would have been otherwise discarded was collected in a sterile container without preservative from each patient immediately after a bowel movement. Rectal swab samples were collected within 5 min after the bowel movement and 3, 6 and 12-27 h later, by inserting a dual Dacron swab moistened with sterile liquid Stuart medium (Becton Dickenson, Sparks, MD) 1-2 cm past the anal verge and rotating the swab gently 360°. Swab samples were stored in the original swab collection container with liquid Stuart medium. Stool and rectal swab samples were stored up to 27 h at 1-8 °C before being frozen at -80 °C.

### Specimen processing for storage conditions analysis

For the storage conditions analysis, each specimen was divided into 15 aliquots to evaluate each of the different storage conditions being tested (Table 1). Aliquots of 0.2 g of stool were prepared in Sarstedt tubes in triplicate for each storage condition. After the samples were subjected to the storage conditions as described, the samples were transferred to an ultralow-temperature freezer.

### DNA isolation, library preparation and sequencing

All specimens were shipped overnight on dry ice to the University of Michigan. Samples were transferred to a 96-well bead plate and then submitted to the University of Michigan Microbial Systems Laboratory for DNA isolation and sequencing. DNA was isolated with a PowerMag Soil DNA Isolation Kit (Mo Bio Laboratories, Inc.) using an epMotion 5075 liquid handling system (Eppendorf). The V4 region of the 16S rRNA gene was amplified and sequenced with a MiSeq (Illumina) as described previously [17]. Fastq files were deposited in the SRA (Bioproject: PRJNA317493).

### Analysis of 16S rRNA gene sequences

The 16S rRNA gene sequence data was processed and analyzed using the software package mothur (v.1.34.4) and MiSeq standard operating procedure described in Kozich et al. [17–19]. After sequence processing and alignment to the SILVA reference alignment (release 109) [20], sequences were binned into operational taxonomic units (OTUs) based on 97% sequence similarity using the average neighbor method. Sample aliquots were removed from the analysis if the number of sequences was below 1000. By calculating θ_YC_ distances (a metric that takes relative abundances of both shared and non-shared OTUs into account) [21] between communities and using analysis of molecular variance (AMOVA) [22] it was possible to determine if there were statistically significant differences between the microbiota of different groups. Principle coordinates analysis (PCoA) was used to visualize the θ_YC_ distances between samples. To assess the effect of storage conditions on community composition, θ_YC_ distances between aliquots of the same sample were compared to θ_YC_ distances between aliquots from different samples using a Wilcoxon test in Prism 6 for Mac OS X (GraphPad Software, Inc.). We also used a Wilcoxon test to compare θ_YC_ distances between swab and stool samples from the same subject and between swab and stool samples from different subjects. The taxonomic composition of the bacterial communities was investigated by classifying sequences within mothur using a modified version of the Ribosomal Database Project (RDP) training set [23, 24].

## Abbreviations

MDRO: Multidrug-resistant organism
OTU: Operational taxonomic unit
